# Attention to the Editor: “Impact of preoperative diastolic dysfunction on short-term outcomes following robotic-assisted minimally invasive esophagectomy (RAMIE)”

**DOI:** 10.1007/s11701-025-02804-5

**Published:** 2025-09-19

**Authors:** Saeed Torabi, Philipp Omuro, Dolores T. Krauss, Sandra E. Stoll, Tobias Kammerer, Georg Dieplinger, Thomas Schmidt, Fabian Dusse, Andrea U. Steinbicker, Christiane J. Bruns, Lars M. Schiffmann, Hans F. Fuchs

**Affiliations:** 1 Medical Faculty of the University of Cologne, Department of Anesthesiology and Intensive Care Medicine, University Hospital of Cologne, Cologne, Germany; 2 Medical Faculty of the University of Cologne, Department of General, Visceral, Thoracic and Transplant Surgery, University Hospital of Cologne, Cologne, Germany

Dear Dr. Albala,

We would like to sincerely thank the correspondents (Roy et al. [1] and Haider et al. [2]) for their thoughtful comments on our recent article *“Impact of preoperative diastolic dysfunction on short-term outcomes following robotic-assisted minimally invasive esophagectomy (RAMIE)”* [3].

We are grateful for their careful review and for raising valuable points that further highlight both the strengths and the limitations of our work.

We fully acknowledge that our cohort predominantly consisted of patients with grade I diastolic dysfunction, with only a very small number of patients presenting grade II dysfunction and none with higher grades. This distribution reflects the typical patient population undergoing RAMIE in our institution, where advanced diastolic dysfunction is rarely encountered. While all four patients with grade II dysfunction developed postoperative atrial fibrillation, we deliberately refrained from drawing firm conclusions from such a small subgroup. Accordingly, we restricted our conclusions to mild dysfunction (grade I) and emphasized in the Discussion that larger, ideally multicenter studies including more advanced dysfunction are required.

We also agree with the concerns regarding statistical power. Although the observed rate of postoperative atrial fibrillation was numerically higher in patients with diastolic dysfunction (25% vs. 18%), the difference did not reach statistical significance. A post hoc power analysis indicated that a sample size of approximately 689 patients would be required to detect this difference with 80% power. Thus, the lack of statistical significance in our cohort should not be interpreted as absence of effect but rather as a limitation of sample size.

With regard to statistical methodology, we acknowledge that the use of χ^2^ testing for rare outcomes such as mortality or anastomotic leakage has limited sensitivity, and that we did not perform multivariable adjustment for confounders such as coronary artery disease, hypertension, diabetes, or body mass index. This was a conscious decision due to the limited number of events, which would have rendered multivariate models unstable and potentially misleading. Instead, we opted to present the comorbidity distributions transparently and to openly acknowledge this as a limitation of our study.

We also recognize the point raised about potential selection bias. In our institution, transthoracic echocardiography (TTE) is performed selectively in patients over 60 years of age or those with cardiac comorbidities as mostly classified bei ASA III. As such, the included cohort indeed represents a higher-risk surgical population, which limits generalizability. At the same time, this reflects real-world clinical practice, where echocardiography is rarely performed in younger, low-risk patients undergoing esophagectomy. Therefore, we believe our findings are particularly relevant to the patient group most frequently subjected to perioperative echocardiographic risk assessment.

Finally, we would like to emphasize that our conclusion is not that diastolic dysfunction is clinically irrelevant, but rather that mild diastolic dysfunction, within the framework of a standardized minimally invasive surgical approach and intensive perioperative care, was not associated with adverse short-term outcomes in our cohort. We fully agree that vigilant monitoring, risk stratification, and targeted perioperative management remain essential, and our study should be interpreted as hypothesis-generating rather than definitive. Nevertheless, this study is surely one of the worldwide largest RAMIE collectives out of a European very high volume center and therefore the best available evidence. We will further evaluate our collective with increasing cases in our prospective database.

In summary, we are grateful for the correspondents’ valuable critiques. Their remarks allow us to refine the interpretation of our data and place our findings in a broader clinical and methodological context. We hope that our study, together with this exchange, will stimulate further prospective and multicenter investigations to clarify the independent prognostic role of diastolic dysfunction in esophagectomy patients.

## Data Availability

No datasets were generated or analysed.
